# Congenital anomalies of kidney and upper urinary tract in children with congenital hypothyroidism; a case-control study

**DOI:** 10.12861/jrip.2015.26

**Published:** 2015-11-30

**Authors:** Parsa Yousefichaijan, Fatemeh Dorreh, Mohammad Rafeie, Mojtaba Sharafkhah, Fatemeh Safi, Mohammad Amiri, Mohsen Ebrahimimonfared

**Affiliations:** ^1^Department of Pediatric Nephrology, School of Medicine, Arak University of Medical Sciences, Arak, Iran; ^2^Department of Pediatrics, School of Medicine, Arak University of Medical Sciences, Arak, Iran; ^3^Department of Biostatistics and Epidemiology, School of Medicine, Arak University of Medical Sciences, Arak, Iran; ^4^Students Research Committee, School of Medicine, Arak University of Medical Sciences, Arak, Iran; ^5^Department of Radiology, School of Medicine, Arak University of Medical Sciences, Arak, Iran; ^6^Department of Emergency Medicine, School of Medicine, Arak University of Medical Sciences, Arak, Iran; ^7^Department of Neurology, School of Medicine, Arak University of Medical Sciences, Arak, Iran

**Keywords:** Congenital hypothyroidism, Congenital abnormalities, Kidney, Urinary tract

## Abstract

**Introduction:** Congenital hypothyroidism (CH) may be significantly associated with congenital malformations. However, there is little evidence on the relationship between renal and urinary tract anomalies and CH.

**Objectives:** The aim of this study was to compare the renal and upper urinary tract anomalies in children with and without primary CH (PCH).

**Patients and Methods:** This case-control study was conducted on 200 children aged 3 months to 1 year, referring to Amir-Kabir hospital, Arak, Iran. One hundred children with PCH, as the case group, and 100 children without CH, as the control group, were selected. For all children, ultrasonography and other diagnostic measures (if necessary) were performed to evaluate renal and upper urinary tract anomalies (ureter and bladder).

**Results:** The frequency of renal and upper urinary tract anomalies among 43 children with primary CH, with 83 cases (72.8%), was significantly higher than the frequency of anomalies among the 19 children in the control group, with 31 cases (27.1%) (OR = 3; CI 95%: 1.6-5.4; *P* = 0.001). Among the anomalies studied, only the differences in frequency of uretero-pelvic junction obstruction (UPJO) (OR = 6; CI 95%: 1.3-28; *P* = 0.018) and hydronephrosis (OR = 22; CI 95%: 5-95; *P* = 0.001) was significant between the two groups.

**Conclusion:** Our study demonstrated that PCH is significantly associated with the frequency of congenital anomalies of the kidneys and upper urinary tracts. However, further studies are recommended to determine the necessity of conducting screening programs for anomalies of the kidneys and urinary tract in children with CH at birth.

**Implication for health policy/practice/research/medical education:**

Better understanding of anomalies associated with the congenital hypothyroidism (CH) could be a critical step towards the identification of the etiology of CH. Since there is little evidence on the relationship between renal and urinary tract anomalies and CH, we evaluated this relationship and we have shown significantly associated of primary CH and congenital anomalies of kidney and upper urinary tract.

## Introduction

Congenital malformation is one of the causes of death in newborns (1). Congenital hypothyroidism (CH) is also one of the common causes of congenital endocrine disorder found in about 1 out of 3000 to 4000 live births (2). Based on the evidence, the prevalence of CH in the United States increased by 73% from 1987 to 2002 (3). As a routine, in many parts of the world, children are evaluated and screened for CH at birth (4). Thyroid dysgenesis is the cause of 85% of the CH cases and other causes of this disorder are related to the dyshormonogenesis (5). Based on the evidence, CH can be associated with congenital malformations (6-11). Cardiac and gastrointestinal anomalies are of the most common congenital anomalies associated with CH (10,12-18). The relationship between extra-thyroidal malformations (ECMs) and CH in newborns may indicate the role of common genetic components in the etiology of CH and its related anomalies (13), while recently, evidence of a genetic link between the incidence of CH and other congenital anomalies have been proposed (12). Based on this evidence, mutations in the genes PAX8 (paired box 8), FOXE1 (forkhead box E1), TTF1 (thyroid transcription factor 1), TTF2 (thyroid transcription factor 2) and TSHR (Thyroid-stimulating hormone receptor) can be associated with thyroid dysplasia and malformations involving the kidney, lung, forebrain, and palate, in addition to interfere with the thyroid follicular cell development (19-23). Better understanding of anomalies associated with the CH, as well as the molecular mechanisms that may be involved in thyroid disorders and its related anomalies could be a critical step towards the identification of the etiology of CH, followed by appropriate management of patients with this disorder (13).

Renal and urinary tract anomalies are common in children and make up about 30% of anomalies at birth (24). These anomalies have a large variety and can involve kidney alone or kidney and urinary tract simultaneously (24). Renal and urinary tract anomalies are of common causes of end-stage renal disease (ESRD), followed by kidney transplantation and dialysis in children, which, based on evidence, are found in 50% of children with ESRD (12,24). Moreover, these anomalies could be a starting point for renal problems in adulthood and development of hypertension, proteinuria, and other problems as well. Several case reports showed that children with CH have disorders in kidney and urinary tract development as well (25-27). However, because of the importance of anomalies associated with CH and studies conducted on renal and urinary tract anomalies in children with CH are small (12), evaluation of these important anomalies in newborns with CH seems reasonable.

## Objectives

The fact that, at present, the children with CH do not pass routine screening for renal and urinary tract anomalies, and due to lack of studies on this subject, we aimed to investigate the renal and upper urinary tract anomalies in children with primary CH (PCH).

## Patients and Methods

### Study patients

This case-control study was conducted on 200 children aged three months to one year, referred to Amir-Kabir hospital, Arak, Iran. One hundred children with PCH were selected as the case group and 100 non-CH children were included in the study as the control group. Positive results of thyroid screening test performed during the first week after birth (blood test taken from the baby’s heel) using serum thyroid function test, including measurement of TSH, T4 or free T4 was confirmed by radioimmunoassay (7,13).

As newborns in Iran do pass routine neonatal screening for thyroid disorders, the case group patients were consisted of newborns diagnosed with PCH in the medical center under study, or patients from other provincial medical centers referred to this hospital to confirm the diagnosis of CH and start the thyroid alternative therapy. After obtaining informed consent from parents or the guardians of children, subjects in the two groups were included in the study using random sampling method, and they were matched for the age (with a standard deviation of ±2) and gender.

A checklist of demographic and clinical data was completed for each child with the assistance of their parents. The checklist included age, sex, history of maternal exposure to hazardous agents during pregnancy, Mothers’ age during pregnancy, mothers’ education, family income and history of CH in the first degree relatives of children.

Mothers’ age at pregnancy was classified in one of the three groups of 20≥, 30-21, and 31≤ years old. Mothers’ education was classified in one of the four groups of 1) elementary school, 2) high school, 3) college, and 4) doctorate and post-doctorate. Family monthly income was classified in one of three groups of less than US $170 (low income), US $170 to US $340 (medium income) and more than US $340 (high income). The history of mothers’ exposure to risk factors during pregnancy was defined as the history of consumption of any amount of cigarettes, alcohol and drugs abuse during the first 3 months of pregnancy. A history of developing CH in the family was defined as the history of CH diagnosed in the first-degree relatives (father, mother, brother or sister).

### Examining of anomalies

Clinical examination and diagnostic procedures were performed on children in both groups for checking renal and urinary tract anomalies, in a way that ultrasound of the abdomen and pelvic was first performed on children by a skilled radiologists, and then, according to preliminary results in suspected cases, further diagnostic measures including Tc99m-DTPA (Diethylene Triamine Pentacaetic acid), voiding cystourethrogram (VCUG), dimercaptosuccinic acid (DMSA) scan and computed tomography (CT) scan of the abdomen and pelvic, were performed as needed for each child.

Renal and upper urinary tract anomalies (ureter and bladder) were examined based on these diagnostic measures; and those children with anomalies developed by events after birth were excluded.

Examined anomalies included renal agenesis, renal dysgenesis (dysplasia, hypoplasia, and cystic anomalies (cystic dysplasia and multicystic dysplastic kidney (MCDK)), ectopic kidney, hydronephrosis, uretero-pelvic junction obstruction (UPJO), Horseshoe kidney, hydroureter, vesico-ureteral reflux (VUR), bladder extrophy, bladder diverticula and urachal anomalies (28,29).

### Ethical issues

1) The research followed the tenets of the Declaration of Helsinki; 2) informed consent was obtained, and they were free to leave the study at any time; and 3) the research was approved by the ethical committee of Arak University of Medical Sciences.

### Statistical analysis

The collected data were analyzed with SPSS version 18.0 (SPSS Inc, Chicago, Ill, USA) and descriptive statistics methods for frequency determination. Categorical data are expressed as number (percentage) and compared with chi-square test. Odds ratios (OR) and 95% CI were calculated for the risk of having problems and the intensity relationship of research variables. A *P* value less than .05 were considered significant.

## Results

The mean age of the all children was 7.6 ± 2.82 years. Among them, 83 (41.5%) and 117 (58.5%) were boys and girls, respectively. Demographic and clinical characteristics of the participants are presented in Table 1. The results showed that the mother’s education (OR = 2.6; CI 95%: 1.6-4.2; *P* = 0.001), family history of CH (OR = 6; CI 95%: 1.3-28; *P* = 0.018) and Mothers’ age at the pregnancy (OR = 0.8; CI 95%: 0.8-0.9; *P* = 0.001) have significant relationship with development of PCH, so that the frequency of PCH was significantly higher in the group of mothers with secondary education (high school), having a positive family history of CH and ≥31 maternal age during pregnancy.

**Table 1:** Demographic data of the study groups

**Variable**	**PCH** ^a^ ** group** **No. (%)**	**Control group** **No. (%)**	**Total** **No. (100%)**	***P *** **value** ^b^
Positive history of cigarette smoking	23 (63.8)	13 (36.1)	36	0.097
Positive history of Alcohol consumption	7 (87.5)	1 (12.5)	8	0.065
Positive history of drugs abuse	4 (80)	1 (20)	5	0.369
Positive family history of CH	11 (84.6)	2 (15.3)	13	0.018
Maternal education				0.001
College	24 (82.7)	5 (17.2)	29	
High school	45 (64.2)	25 (35.7)	70	
Elementary school	27 (32.1)	57 (67.8)	84	
Doctorate and post-doctorate	4 (23.5)	13 (76.4)	17	
Maternal age at pregnancy (year)				0.001
≥20	5 (27.7)	13 (72.2)	18	
21-30	34 (33)	69 (66.9)	103	
≤31	61 (77.2)	18 (22.7)	79	
Family income				0.229
Low^c^	8 (38)	13 (61.9)	21	
Medium^d^	50 (56.1)	39 (43.8)	89	
High^e^	48 (53.3)	42 (46.6)	90	

^a^Children with primary congenital hypothyroidism. ^b^ *P* value less than 0.05 were considered significant. ^c^Family monthly income less than US $170. ^d^ Family monthly income US $170 to US $340. ^e^ Family monthly income more than US‏ $340.

Of 114 (100%) renal and urinary tract anomalies diagnosed among 43 children in the case group and 19 children in the control group, 83 (72.8%) and 31 (27.1%) cases of anomalies were associated with children in the case and control groups, respectively. Accordingly, the frequency of renal and urinary tract anomalies in the children with PCH was significantly more than non-CH children (OR = 3; CI 95%: 1.6-5.4; *P* = 0.001).

**Figure 1:**
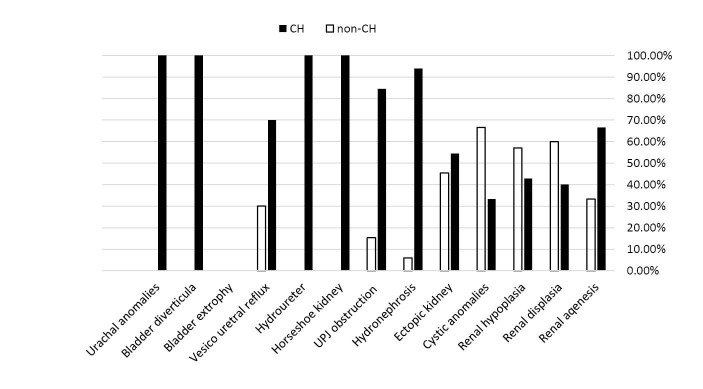
The frequency of renal, ureter and bladder anomalies in children with and without congenital hypothyroidism

Among the anomalies studied, only the differences in frequency of UPJO (OR = 6; CI 95%: 1.3-28; *P* = 0.018) and hydronephrosis (OR = 22; CI 95%: 5-95; *P* = 0.001) were significant between the two groups, thus the frequency of UPJO and hydronephrosis, with 11 (84.6) and 31 (93.9%) cases, respectively, in the children with PCH, was significantly higher than the control group, and there were no significant differences in frequency of other anomalies between the two groups (Table 2, Figure 1).

**Table 2:** The frequency of renal, ureter and bladder anomalies in children with and without primary congenital hypothyroidism

**Anomalies**	**Frequency of anomalies in the PCH group** **No. (%)**	**Frequency of anomalies in the control group** **No. (%)**	**Total** **No. (100%)**	***P *** **value**
Renal agenesis	6 (66.6)	3 (33.3)	9	0.498
Renal dysgenesis				
Dysplasia	2 (40)	3 (60)	5	<0.05
Hypoplasia	3 (42.8)	4 (57.1)	7	<0.05
Hypodysplasia	0 (0)	0 (0)	0	-
Cystic anomalies				
Cystic dysplasia	3 (66.6)	2 (33.3)	5	<0.05
MCDK	1 (25)	3 (75)	4	<0.05
Ectopic kidney	6 (54.5)	5 (45.4)	11	<0.05
Hydronephrosis	31 (93.9)	2 (6)	33	0.001
UPJO	11 (84.6)	2 (15.3)	13	0.018
Horseshoe kidney	1 (100)	0 (0)	1	<0.05
Hydroureter	2 (100)	0 (0)	2	0.497
VUR^3^	14 (70)	6 (30)	20	0.097
Bladder extrophy	0 (0)	0 (0)	0	-
Bladder diverticula	1 (100)	9 (0)	1	<0.05
Urachal anomalies	3 (100)	0 (0)	3	0.246
Total No. (%)	83 (72.8)	31 (27.1)	114	0.001

Abbreviations: MCDK,multicystic dysplastic kidney; UPJO, uretero pelvic junction obstruction; VUR, vesico-ureteral reflux.

The results of correlation between risk of renal congenital anomalies and the history of maternal exposure to hazardous agents in the first 3 months of pregnancy are presented in Table 3. The results showed that the frequency of renal dysplasia in the group of mothers with alcohol consumption(OR = 21; CI 95%: 2.9-149.8; *P* = 0.013), the frequency of renal hypoplasia in the groups of smokers (OR = 32; CI 95%: 3.7-280.5; *P* = 0.001), and the frequency of renal agenesis in cigarette consumption (OR = 19.5; CI 95%: 3.8-98.8; *P* = 0.001), in the group of mothers without a history of alcohol abuse (OR = 19.5; CI 95%: 3.8-98.8; *P* = 0.001) and in the group with a history of drug abuse during pregnancy (OR = 0.4; CI 95%: 0.2-0.9; *P* = 0.001) is significantly higher, while there is no significant correlation between other anomalies, with the history of smoking, alcohol and drugs abuse at the first three months of pregnancy.

**Table 3:** The relationship between maternal exposure to hazardous agents during the first 3 months of pregnancy and the risk of congenital renal anomalies

**Hazardous agents**	**Dysplasia (n = 5)** **No. (%)**	***P*** ** value**	**Hypoplasia (n = 7)** **No. (%)**	***P*** ** value**	**Agenesis (n = 9)** **No. (%)**	***P *** **value**
Cigarette smoking	2 (40)	0.221	6 (85.7)	0.001	7 (77.7)	0.001
Alcohol consumption	3 (60)	0.013	1 (14.2)	0.252	2 (22.2)	0.044
Drugs abuse	0(0)	<0.05	0 (0)	<0.05	5 (55.5)	0.001

## Discussion

The main objective of our study was to examine those extra-thyroidal malformations which are less studied in children with CH. Based on our results, the frequency of renal, ureters and bladder anomalies in children with PCH was significantly higher than children without CH. Among the anomalies under study, only the frequency of hydronephrosis and UPJO in children with PCH was significantly more than children without CH, and there were no significant differences in frequency of other anomalies between the two groups.

Based on the New York state congenital malformation registry, Kumar et al (12), among 980 children with CH and 3 661 585 non-CH children born between 1992 and 2005 in the United States, showed that children with CH are significantly prone to comorbidity of renal and urinary tract anomalies, particularly hydronephrosis and renal agenesis. Based on neonatal screening in four northeastern regions of Italy, Cassio et al (7), examined 235 newborns with CH selected from among 745.801 newborns screened in terms of congenital malformations. Authors showed that the prevalence of congenital anomalies in children with CH and in the general population is 9.4% and 1.8%, respectively. Based on this study, the prevalence of internal urogenital anomalies in children with CH and in the general population of children was 0.43% and 0.11%, respectively, which there was no significant difference between the two groups. In the two case reports, Satomura et al (25), and Jeha et al (27), introduced newborns that were diagnosed with glomerulocystic kidney disease (GCKD) and autosomal recessive polycystic kidney disease (ARPKD), respectively, in addition to having CH. Reddy et al (30), in 2010, in India, reported no cases of urogenital anomalies among 70 children with CH. Most of the studies on congenital anomalies associated with CH have studied extra-urinary tract malformations and less studies are found to be conducted on the evaluation of renal and urinary tract anomalies in children with CH. In a retrospective review, Gu et al (31), studied ECMs among 1520 newborns. Based on this study, the incidence of ECMs in newborns with CH (14.6%) was significantly higher than the general population.

Olivieri et al (13), examined the incidence of congenital anomalies among 1420 children with CH born between 1991 and 1998 in Italy. In this study, the prevalence of congenital malformations in children with CH (8.4%) was 4 times the incidence in the general population (1.2%). Also, the results of this study showed that CH is significantly correlated with the risk of cardiac anomalies (more than any other anomalies), nervous system and ophthalmic anomalies.

In a cross-sectional study on 76 children with PCH, Kreisner et al (10), demonstrated that the frequency of major congenital malformations in children with CH (13.2%) is significantly more than non-CH children.

In the present study, it is indicated that the incidence of dysplasia is significantly correlated with the history of alcohol consumption, hypoplasia is correlated with the history of smoking, and renal agenesis is correlated with the history of smoking and drug abuse by the mother during the first three months of pregnancy.

In a study conducted on 483 newborns with renal malformations and 719 newborns with other urinary tract anomalies, apart from renal anomalies, Källen (32), reported a moderate significant correlation between maternal smoking history and the risk of congenital renal malformations including agenesis, hypoplasia and dysplasia. However, according to this study, association between the history of smoking and alcohol consumption by the mother during pregnancy and the risk of urinary tract anomalies were not significant. In a study conducted on 118 children with congenital anomalies of the urinary tract, Li et al (33), found a strong association between smoking during pregnancy and the risk of congenital urinary tract anomalies. Additionally Clarren and Smith (34) demonstrated that the incidence of renal hypoplasia and dysplasia could be related to the fetal alcohol syndrome. Likewise in a study of the fetus of 13 smoking mothers and 13 non-smoking mothers in weeks 22 to 28 and 33 to 38 of pregnancy period using magnetic resonance imaging (MRI), Anblagan et al (35), demonstrated that maternal smoking during pregnancy has a significant relationship with the growth retardation of fetal brain, lungs and kidneys.

When a teratogenic agent may be involved in causing congenital malformations, determination of the exact relationship between these agents and risk of congenital anomalies, in order to minimize the prevalence of these anomalies and short- and long-term effects is necessary and very important (32). Based on the evidence, smoking could be involved in the development of urinary tract anomalies through interfering with the ureteral bud development and developmental relation between ureteral bud and metanephric mesenchyme (32). However, the exact mechanism of the association of smoking, alcohol and other drugs abuses with the increased risk of renal and urinary tract anomalies is currently unclear (32,35). According to our study and other studies, it seems that smoking, alcohol and other drugs abuses are associated with the increased risk of renal anomalies in newborns. However, due to the paucity of studies in this field, the existence of some relevant controversial results and also the importance of identifying teratogenic factors associated with renal and urinary tract anomalies in newborns, future research in this area, particularly in relation to finding mechanisms of the relationship between teratogenic agents such as cigarettes and alcohol consumption with renal and urinary tract anomalies is recommended. Since only the relationship between the any amount of cigarette smoking, alcohol consumption and any drug abuse in the first 3 months of pregnancy and the renal and urinary tract anomalies was studied in the present study, it is suggested that the future studies in this area to be conducted by considering the relationship between the amount of cigarettes, alcohol and drugs consumed by the mothers and the type of drugs used during the nine months of pregnancy, and the risk of congenital anomalies of the kidney and urinary tract.

Given the evidence of the involvement of a common genetic component to the development of CH and congenital anomalies associated with it (13,19-23), and according to numerous clinical evidence obtained about significant association between congenital anomalies and CH (7,10,12-18,31), the association between CH and congenital anomalies can be considered almost inevitable. However, due to the following reasons, continuing research in this area, particularly in relation to anomalies that were less studied, (such as renal and urinary tract anomalies conducted in the present study) is proposed for future studies.

1) Examination of the correlation between CH and different type of congenital anomalies can be helpful in better determining their possible common etiology (13).

2) While there are numerous evidences about the association of some congenital anomalies, especially cardiac anomalies with CH (10,12-18,30), however, evidence about the correlation of some prevalent congenital anomalies, including renal and urinary tract anomalies with CH is little (12).

3) Further studies in the future and then reaching a comprehensive conclusion about the relationship between each of the congenital anomalies and CH, can be helpful in better management of patients, planning for screening and preventive treatments to improve the prognosis in the children.

## Conclusion

Our study demonstrated that PCH is significantly associated with the frequency of congenital anomalies of the kidneys and upper urinary tracts. However, further studies are recommended to determine the necessity of conducting screening programs for anomalies of the kidneys and urinary tract in children with CH at birth.

## Limitations of the study

Some of limitations of our study were the small sample size, lack of study of the extra-urinary tract malformations (with respect to the health center facilities under study) and failure to evaluate the frequency of renal and urinary tract anomalies based on the cause of CH. It is recommended that future studies in this area to be conducted on a larger sample size, taking into consideration the CH causes, and other extra-urinary tract malformations.

## Acknowledgments

The research team wish to thank vice chancellor of research for their financial support and also children and their parents who contribute in this research.

## Ethical considerations

Ethical issues (including plagiarism, data fabrication, double publication) have been completely observed by the authors.

## Conflicts of interest

The authors declared no competing interests.

## Authors’ contribution

PY; participated in the design of the study, served as the lead author of the manuscript. MSh; performed the data collection, wrote the first draft of the manuscript and statistical analysis. FD, MA, ME, FS; wrote some parts of the draft. MR; participated in the design of the study and statistical analysis. All authors read and approved the final manuscript, and no payment was given to anyone to produce the manuscript.

## Funding/Support

This study was financially supported by Arak University of Medical Sciences (Grant # 806).
